# The enhancement of academic integrity through a community of practice at the North-West University, South Africa

**DOI:** 10.1007/s40979-022-00115-y

**Published:** 2022-08-18

**Authors:** Anné Hendrik Verhoef, Mariette Fourie, Zander Janse van Rensburg, Henk Louw, Mianda Erasmus

**Affiliations:** 1 School of Philosophy, North-West University, Potchefstroom, South Africa; 2 Academic Programmes, North-West University, Quality Enhancement Office, Potchefstroom, South Africa; 3 Writing Centre, North-West University, Potchefstroom, South Africa; 4 School of Psychosocial Health, North-West University, Potchefstroom, South Africa

**Keywords:** Academic integrity, Academic success, Appreciative inquiry, Community of practice, Covid-19, Higher education, Teaching, Learning and assessment

## Abstract

This article was motivated by the need to academically frame and share the response of the North-West University (NWU) to the perceived increase of academic dishonesty during Covid-19. Within the ambit of the online (hybrid) teaching and learning approach that became dominant during the Covid-19 pandemic, the NWU established a Community of Practice for Academic Integrity (CoPAI) to enhance Academic Integrity (AI) in a holistic manner. By critically discussing the NWU’s response through their CoPAI, the lessons learned, and strategies developed in the process, the NWU can hopefully assist other Higher Education institutes to progressively enhance AI in the future. This is important, because many contextual shifts in teaching and learning approaches, pedagogy, assessment, and the application of technology, that were enforced in an online mode of delivery during the pandemic, will prevail in future.

In writing this article, we focused on contextualising the NWU CoPAI within current literature on community of practice (CoP) and Academic integrity (AI) and emphasising the unique strategy and holistic nature of this CoPAI. The establishment of the CoPAI is discussed within the appreciative inquiry as methodological framework. This methodology is commonly used by CoPs, but it is particularly relevant to the CoPAI since CoPAI sought answers to all the AI questions that presented itself due to disruptions in the higher education landscape. The appreciative inquiry method allowed for the opportunity to find some answers in a holistic manner. Some of these answers or insights gained through the activities of CoPAI is further discussed in the latter part of the article. In conclusion, some of the outcomes and shortcomings of CoPAI at the NWU are highlighted.

The main finding of this article concluded that the establishment of a CoPAI can enhance AI at HE institutions in a holistic manner. The applicability, relevance, and success of this CoPAI was realised through its holistic approach which included the valorisation of institutional aspects, the engagement and empowerment of lecturers, and the engagement and empowerment of students. This novel and unique approach to promote AI in HE could fill the existing knowledge gap in the South African context, where the establishment of a CoPAI, the application of appreciative inquiry as methodology, and the inclusion of a holistic approach are still absent. It might however also be an example for other HE institutions to follow globally.

## Introduction

In acknowledging the paucity of research on the formation of a CoP and its value for AI as such, this article indicates how a CoP at the North-West University in South Africa managed to enhance AI at this institution in a holistic and unique way. The term COP is taken from Lave and Wenger’s (1991) work who first coined the term, ‘Community of Practice’, in their seminal text, *Situated Learning: Legitimate peripheral participation*. It focused on situated learning and challenged the conventional, cognitive understanding of the time that learning is internalised knowledge transmitted from teacher to pupil: “We suggest that learning occurs through centripetal participation in the learning community of the ambient community” (Lave and Wenger 1991:100). This understanding of learning as a “trajectory into a community”, rather than a handing down of facts, became the central theme of Lave and Wenger’s work (Mercieca 2017:8).

The article first explores, through a literature review, the possible roles of CoPs as a tool or approach to enhance AI at institutions of HE. The uniqueness of the CoPAI at NWU regarding its strategy, holistic nature, and inclusion of student voices, is further highlighted in this section. In the second part of the article, background is given to the formation of this CoPAI, its strategy, and its functioning at the NWU. The third part of the article explores the insights gained and knowledge created through the CoPAI forums as part of the appreciative inquiry methodology that was followed. In the fourth part of the article, the concrete outcomes of the different activities of CoPAI at the NWU will be expounded by discussing some action plans that were developed, the work on Standard Operating Procedures (SOP) for AI in the teaching and learning environment, and other initiatives thus far. This is all part of the “dream, design and delivery” phase of the appreciative enquiry approach that has been followed with and by the CoPAI. The conclusion of the article then highlights the main contribution of this article in the field of academic integrity, by indicating the importance, value, and relevance of a CoPAI for the enhancement of AI at HEIs through the example of the CoPAI at NWU in South Africa.

### The possibilities of communities of practice for enhancing academic integrity

A number of articles have been published on academic integrity since the Covid-19 pandemic erupted at the end of 2019 and caused the global higher education sector to switch to online teaching, learning and assessment (TLA). Many of these articles focused on students’ experiences of remote education and perceptions of online cheating (Khan et al., 2021; Walsh et al., 2021; Meulmeester et al., 2021; Meccawy et al., 2021; Reedy et al., 2021a), online assessments and exams (Gorgani et al., 2021; Cahapay, 2021), and contract cheating (Usick & Stoesz, 2021). Some of these themes have already been researched prior to the pandemic (e.g., Waghid et al., 2019; Draper et al.,  2017; Awdry and Newton, 2019; Newton, 2018), and although these findings remain relevant to a large extent, the situation has changed radically with the pandemic, and new solutions had to be found.

Consequently, since the onset of Covid-19 there have been several conferences on academic integrity (e.g., the European Conference on Academic Integrity and Plagiarism of 9–11 June 2021),^1^ research reports, and the publication of guidelines and strategies on how to ‘regain’ academic integrity during (especially) online assessment. Various articles appeared in *the International Journal for Educational Integrity*, for example. Two significant publications in this regard are the special issue of the *Canadian Perspectives on Academic Integrity* (Vol 4(2) 2021)^2^ and the work done by the University of Calgary with their “Integrity Hour” (Eaton et al., 2021). The importance and role of a CoPAI is a salient theme in these publications.

In the South African context, various articles were also published on academic integrity since the outbreak of Covid-19. For example, Verhoef and Coetser (2021) explored student voices about remote online assessments, Mutongoza (2021) focused on the impetus for cheating, and Verhoef et al. (2020) examined the disruption of the pandemic regarding our existence and how this can also be an impetus for cheating and academic dishonesty. The focus of Baboolal-Frank (2021) is not so much on academic integrity, but rather on learning processes and methodologies during emergency remote learning. An exploration from a South African perspective of the importance and role of a CoPAI as a preventative measure, is lacking in recent publications, however.

The possibilities of COPs for enhancing AI attracted research attention internationally. The University of Calgary, for example, initiated an “Integrity Hour” as part of their CoP (Eaton, 2020). The purpose of this CoPs is “to provide support to those working in higher education who were experiencing changes in the nature of academic integrity breaches, along with a general increase in misconduct cases” (Eaton, 2020:3). The guide for this CoP explains how it functions as a support group for academics, without having the wide scope of the CoPAI of the NWU (which is discussed in this article).

In an article “Reflections on the First Year of Integrity Hour: An Online Community of Practice for Academic Integrity” (Eaton et al., 2021), eight academic lecturers shared their experiences and perspectives of Integrity Hour as CoP in the form of a micro-essay. The support group nature of this CoP became clear in statements from participants that it is a safe space for everyone who participates, that it nurtured professional growth, that learning took place from dedicated, like-minded professionals whose focus is academic integrity, that they could test their own ideas in a safe forum, that enhanced their ability to advocate for a similar vision of Academic Integrity, and that it provided them with the inspiration and energy to making a positive impact (Eaton et al., 2021:6–12). These remarks confirm the importance of a sense of community that must be part of communities of practices. The research of Nistor et al. (2014), for example, indicated the “significant mediating effect of sense of community for … knowledge sharing acceptance” (2014:270). This became visible from some participants in the Eaton et al. (2021) article who reported how the CoP helped them on a practical level to support their students and to include early introduction of the importance of academic integrity, to talk about viable solutions, and to implement processes and procedures proactively. These are all extremely important aspects of a CoP, and it explains why it is still flourishing after a year. The scope of this CoP is, however, is limited in comparison with the NWU’s CoPAI and their impact will be subsequently limited.

Jamieson’s article “Keeping a Learning Community and Academic Integrity Intact after a Mid-Term Shift to Online Learning …” (2020) focused not so much on a CoPAI, but rather on the role of a CoP in preserving “both the quality of our students’ learning experience and academic achievement following a transition to remote meetings, remote exams …” (Jamieson, 2020:2768). The value of this approach at the University of Alberta lies in the fact that students were included in the CoP and that it was a more holistic approach than a mere CoPAI. The shortcoming is that it was very focused on a specific course (Chemical Engineering) and did not make provision for participation by the rest of the institution (or at least did not report on such inclusion in the article).

Krautloher et al. (2021) argued that CoPs are a better way for professional development of academics than professional development sessions. The CoP they created focused specifically on improving assessment practices (like Interactive Orals or IOs) to make it more sustainable for online delivery, whilst maintaining academic integrity. Their CoP met on a weekly basis to “discuss the subject designs of the subjects involved in the pilot and developed a range of resources to administer the IOs successfully” (Krautloher et al., 2021:1). Their CoP thus included academic integrity, although it was not specifically focused on it. The main point of this article, however, is to illustrate how a CoP can be much more effective at developing academics than traditional professional development sessions.

The article of (Scheele et al., 2021) also focused on the role of a CoP in subject design and assessments, and specifically interactive oral assessments (IOAs). This was part of a broader institutional project to improve online assessment practice. It was not a CoPAI per se, because this CoP’s main aim was to design online assessments that promote academic integrity and “reflect authentic graduates’ practice” (Scheele et al., 2021:93). Like Krautloher et al. (2021), it stresses the value and role of CoPs for learning design and professional development. The conclusion of the article highlighted the value of a CoP in supporting academics to embed a new assessment approach across the university courses. The scope of this CoP is however also not as inclusive as the CoPAI at NWU, because it focused mainly on the lecturers and assessments, with specific focus on online assessment practices.

In their article “A community of practice approach to enhancing academic integrity policy translation: a case study”, (Reedy et al., 2021b) do not address a CoPAI in the strict sense of the word, but rather a CoP with a specific task and focus, namely, to translate complex and often ambiguous academic integrity policy into accessible academic integrity resources. The aim was to make it intelligible to staff and students so that the academic staff can consistently enact the policy. The CoP comprised academic and professional staff as a small community of committed grass-roots practitioners argued for greater understanding of the contribution on grass-root levels of academic and professional staff to the translation, interpretation, and enactment of the academic integrity policy. It illustrated how academics (members of the CoP) used their own discretion to understand and implement an academic integrity policy. This CoP thus aimed to improve academic integrity by making the academic integrity policy more understandable and applicable, and thereby had an impact on the university community at large. The importance for a CoPAI to engage with the translation and interpretation of academic integrity policies at institutions is thereby highlighted. This is also an important aspect and task of the NWU CoPAI as will be explained later.

This brief literature review of recent publications on academic integrity (since Covid-19), with emphasis on the ways in which CoPs can enhance AI, indicates the importance of CoPs in this context, but also its limited use or implementation so far. In the South African context, a gap regarding COPs for AI in the literature exists, whereas in the international context, the literature has a limited focus on and engagement with the influence of CoPs regarding the enhancement of AI in a holistic manner.

As clarified in the next sections of this article, the CoPAI at the NWU has a more inclusive scope, engagement, and influence than the CoP’s examined in the literature above. The NWU CoPAI, for example, has a student constituent, and it focuses holistically on academics (assessment, curriculum design, etc.), students (needs, impetuses for cheating, etc.) and the university as an institution (policies, student judicial services, etc.). This will be the focus of the next section where the need for such a holistic approach will be motivated. It should be noted here though, that this holistic approach to promote AI in HE is a unique and novel approach in the South African context. No other universities in South Africa have established a similar CoP and there is also a gap in the literature in the SA context regarding establishment of a CoPAI. Specifically, the application of appreciative inquiry as methodology, and the inclusion of a holistic approach within the context of enhancing academic integrity at HE institutions in South Africa, are absent.

### The formation, strategy and functioning of the NWU CoPAI

The CoPAI at the NWU was the result of two developments. On the one hand, professional development initiatives have always been undertaken by faculties and support departments at the NWU to promote AI at the institution. These initiatives focused on various TLA aspects – for example, the Centre for Teaching and Learning (CTL) at NWU engaged in critical conversations about plagiarism with lecturers and students in 2018 already. These conversations on academic integrity extended into 2020 and increased during COVID-19, when the switch to “emergency remote teaching” (Hodges et al., 2020:1) took place. AI became an urgent and pertinent matter with the online TLA during 2020 and 2021.

On the other hand, the discussions on AI gained much more prominence and became extensive at the NWU after the annual NWU institutional public forum in May 2021. This forum, entitled, ‘Cheating, dishonesty, and plagiarism with online TL. What are the students saying? Can we fundamentally change it?’ was organised by the NWU School of Philosophy. It brought together all the relevant stakeholders of the NWU on AI, and afterwards staff from the Quality Enhancement Office, the School of Philosophy and the Centre for Teaching and Learning (CTL) recognised that there was a need to engage in further critical conversations regarding AI in a holistic, nuanced, and multi-disciplinary manner at the NWU.

It was against this background that the need for a collaborative and integrated approach to enhance AI at the NWU became eminent. The consensus was that the focus should not only be on academic misconduct, but that the conversation should go beyond that incorporating all aspects of AI. This initial small group had decided then to invite all interested academics and support staff for a conversation on AI, and specifically on the possible establishment of a CoPAI at the NWU. The idea of a COP for AI was discussed at the first CoPAI forum, entitled “Towards an Academic Integrity CoP at NWU”, and was well received by all who attended (about a hundred people) resulting in the establishment of the CoPAI was. This first CoPAI forum thus served as the founding event of the NWU CoPAI, where the purpose, strategy and functioning of CoPAI was also decided upon. Therefore, in short, the NWU CoPAI was established due to the need to rethink education and assessment in the ambit of online TLA – with a perceived increase in academic dishonesty – as exacerbated by the Covid-19 pandemic. Since its inception, CoPAI was inclusive of all programmes, faculties, and departments (support and academic) on all campuses of the NWU, and it was officially driven by the Centre for Teaching and Learning, and the Quality Enhancement Office at NWU.

Although the NWU Registrar and the Deputy Vice-chancellor for teaching and learning at the NWU both supported this initiative, a bottom-up approach was still followed. For example, the purpose, strategy, and approach of CoPAI were formulated at the first CoPAI forum; as it was not considered to be a task or mandate of management (thus, it was not a top-down process). Furthermore, participation in CoPAI was voluntary and there was no official or closed membership list. Everyone at the NWU was invited to all the CoPAI activities. Persons who attended previous activities were followed up for feedback (especially to determine further needs) and to market future CoPAI events.

At the first CoPAI forum it was decided that the purpose of CoPAI would be to enhance AI at the NWU, by means of:

- sharing best practices through appreciative inquiry as research methodology,
- creating and sharing knowledge in the form of research and practical (teaching and learning) outputs,
- fostering national and international collaboration on academic integrity, and
- establishing a CoPAI identity at the NWU.

In more practical terms, this means that CoPAI strives to:

- create opportunities (e.g., forums) where lecturers, students and support departments can share best practices in a free and open way,
- deliver some practical outputs and guidelines, as well as research outputs on AI (these deliverables are discussed as part of the methodology section, specifically referring to the design and delivery phase of the 4D model following an appreciative inquiry methodological approach),
- collaborate with national and international scholars through sharing of best practices, and by inviting them as speakers to the CoPAI forums,
- establish a CoPAI identity through various marketing strategies. To this end, CoPAI has established its own CoPAI website^3^ and a CoPAI Facebook page^4^ to enhance collaboration and engagement amongst its community members. An initial report in the university newsletter^5^ was also published on the NWU webpage.

**Fig. 1:**
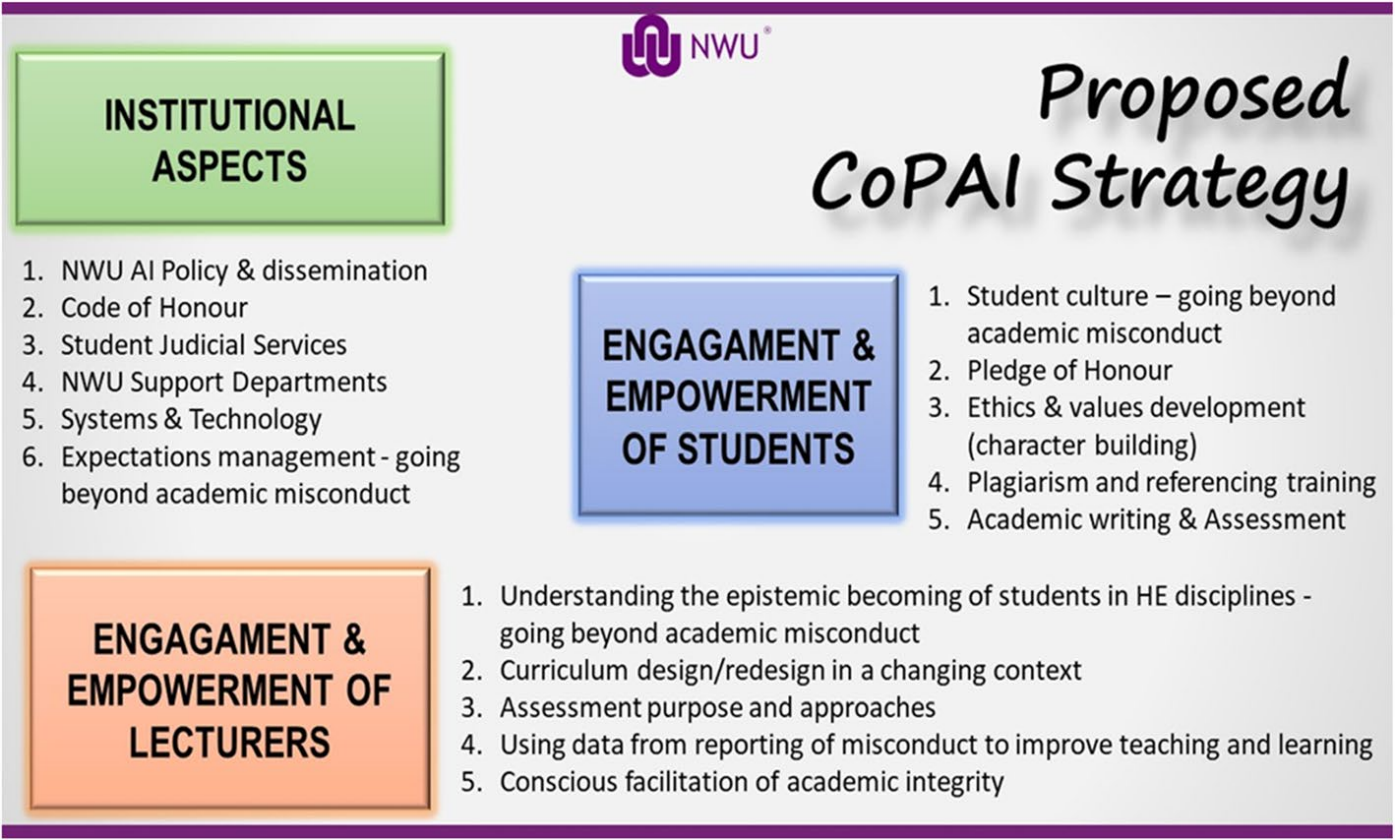
The CoPAI three-point strategy

A crucial aspect of the CoPAI strategy is to address AI at the NWU in a holistic way. This approach includes three main aspects, namely 1) the valorisation of institutional aspects, 2) the engagement and empowerment of lecturers, and 3) the engagement and empowerment of students. See Fig. 1 below for a summary of these three main strategic focus points of CoPAI. These three strategic focus points determine the way CoPAI functions and will be discussed in more detail below.

#### Institutional aspects

At an institutional level, various aspects were identified to address AI at the NWU. The first is the NWU Policy on Academic Integrity.^6^ Although this policy exists and is relatively up to date, an educative focus on the dissemination of this policy is urgently needed.

The second aspect that was identified is the function and role of the Student Judicial Services (SJS) at the NWU, which deals with the disciplinary aspect of academic misconduct. One crucial aspect identified regarding SJS was the need for a consistent approach across all faculties in dealing with academic misconduct. This became a crucial point of debate amongst participants in various CoPAI forums and the consensus was that a much more robust, consistent, fast, and fair process is needed for dealing with relevant breaches at the NWU. The developmental aspect of disciplinary actions also enjoyed prevalence during discussions. In addition, it was emphasised that there was a need for data on academic misconduct and the reasons which students identified for such misconduct. Based on this, it also became evident that the institution needs to adopt a data-driven approach in making decisions for driving academic integrity at the institution. All these aspects were identified through the CoPAI forums as urgent tasks or aspects for intervention by CoPAI at an institutional level to enhance AI.

A third aspect identified to address AI at the NWU at an institutional level was the need for collaboration among and between NWU support departments and academic faculties. This must take on a much more practical level to ensure quality assurance, the integrity of the institution, as well as the integrity of its qualifications. For example, from a systems and technology perspective, the apt use and interpretation of Turnitin reports were discussed. The effective use of a variety of software systems in combination with the university learning management system (LMS) in an online environment, were highlighted in the discussions. Academic misconduct was also discussed as a systemic issue, with the question of how to address this through academic acculturation and education through the compulsory Academic Literacy modules at the NWU, and the NWU code of honour.

#### Engagement and empowerment of lecturers

The second main strategic focus of CoPAI that was decided on, was the engagement and empowerment of lecturers to enhance AI at the NWU. This was one of the biggest needs communicated by the participants at the CoPAI forums, and various aspects were identified to engage and empower lecturers, such as:

- The training of lecturers to understand the epistemic becoming of students in HE. This is crucial to move beyond academic misconduct and help to support and educate students to enhance AI.
- The importance of curriculum design and redesign in a changing online educational landscape. The provision of specific continuous professional development initiatives was identified at this point.
- The design of online assessments. This includes the need to redefine the purpose of assessment as assessment for learning, and to rethink alternative assessment approaches. At various CoPAI forums, the topic of assessments was spurting important conversations and participants shared on this point very practical tips and best practices.
- With reference to systemic issues identified, participants believed that data from reporting academic misconduct must be valued to improve teaching and learning. Suggestions to engage in scholarship of teaching and learning (SoTL) research at the institution could also improve teaching, learning and assessment strategies. Furthermore, the need was identified to consciously facilitate academic integrity on all platforms, especially in classrooms and by all lecturers at the NWU.

#### Engagement and empowerment of students

From the beginning, the CoPAI strategy was holistic in nature and therefore strongly focused on engaging and empowering students to enhance AI at the NWU. The emphasis of this point was to educate students to avoid academic misconduct, to help them create a culture of academic integrity, and to hear from them what their needs are. Although students were also invited to the general CoPAI forums, they did not participate very actively. To create a platform where the students could voice their concerns about AI, a student constituent of CoPAI was formed. This gave students the freedom to voice their concerns, and it also gave the CoPAI leaders the opportunity to listen to their specific needs and to communicate with the different stakeholders, such as SJS or lecturers.

The first student constituent CoPAI forum is discussed in the next section of the article and gives an indication of the value of this platform for enhancing AI at NWU. What needs to be mentioned in terms of engagement and empowerment of students, included:

- The importance of understanding the student profile and associated challenges by lecturers. In South Africa today, the socio-cultural diversity of students is posing various challenges for academic integrity and lecturers should be aware of this.
- The necessity to have an effective and efficient pledge of honour. Although this exists as part of the NWU AI Policy, it is not implemented at the university.
- Students confirmed that the development of students’ ethics and values are important and that the university should address this through various means (e.g., the compulsory foundation educational modules). A student culture that goes beyond academic misconduct, needs to be evident.
- Students asked for a more rigorous approach to plagiarism and reference training. This includes training in proficient academic writing, and in approaching and engaging with assessments.

### Insights gained through CoPAI forums as part of an appreciative inquiry approach

The formation, strategy and functioning of the CoPAI at the NWU, as discussed above, clearly indicates that the aim of CoPAI was to find solutions to problems presented by the pandemic (to enhance AI at the NWU) in collaboration with all the different stakeholders. CoPAI is not a professional development activity or programme offered by the NWU, but rather a community that shares and discusses best practices to find real-life solutions. This approach to problem-solving is best described as an “appreciative inquiry methodology”. Appreciative inquiry is an innovative problem-solving approach, instilling self-determined change that focuses on solutions rather than problems only (Moore, 2021). It offers researchers a means to go beyond the “ordinary” thinking performed in “normal science”. CoPAI embraced this approach from the beginning and followed it with all its initiatives because we realised that we had to find answers among ourselves for the new and unique challenges for AI within the changing (online and hybrid) HE landscape.

CoPAI further adopted an appreciative inquiry as a methodological approach because of its strengths-based change approach and its affirmative approach, which assumes that each social system has a positive core of strengths. In other words, we share the theoretical assumption of an appreciative inquiry that there are some strengths in our CoP which must be discovered and developed to bring about change. In this theoretical framework the emphasis is on appreciation, inquiry, and wholeness. CoPAI followed these three notions in their activities and research (such as this article) as follows:

- The term *appreciative inquiry* already implies that appreciation is crucial in this form of methodological approach. In this approach, *appreciation* means that the COPAI values and recognises its members’ contributions as a point of departure and engages with it not to criticise (or dismiss) it, but to build on its strengths. These collated strengths of the community then become then the foundation for possible positive change. Within the activities of CoPAI, appreciation was consistently emphasised in the sharing of best practices for example. CoPAI thereby attempted not only to find possible solutions, but to build relationships, develop a sense of community (belonging), and strengthen commitment to the process by hosting various discussion forums. This created an open and honest environment where participants could engage in conversations about the realness of academic misconduct, and in finding solutions together.
- In an attempt to find solutions together, appreciative inquiry does not stop at appreciation, but inquires what has been shared. The auspice of inquiry drives curiosity and a desire to discover. As part of this method, inquiry thus is to ask questions to learn or discover from one another (again, not to dismiss others), and to collaboratively identify a shared solution and/or vision. Inquiry, as part of the appreciative inquiry methodological approach, entails specific techniques and operational steps that bring about positive change. This inquiry process is driven by the 4D cycle or model which is explained below. CoPAI adopted this 4D model to thematically analyse the contributions of members throughout the various forum discussions (as deliberated on below), and to discover some solutions to the AI problems. This is also the method followed in writing this article firstly as an appreciation of CoPAI, and then as an attempt to inquire what strengths emerged and the specific solutions or changes that crystallised from that.
- Wholeness is important as part of the appreciative inquiry methodological approach because it encourages participation on all levels. Such participation is needed to discover strengths on all levels and to develop possible solutions on all levels but especially for the whole of the entity or institution such as a university. In this vein, CoPAI adopted a holistic strategic approach, as discussed above. This includes institutional aspects, the empowerment of lecturers, and the empowerment of students. The participation of CoPAIs ensures this wholeness through its holistic approach that includes everyone on all levels of the institution, including all management levels, all academic and support staff, and students across all faculties and campuses.

### The appreciative inquiry 4D model

The first step is to define and contextualise the strategic focus of inquiry i.e., AI through a CoPAI. This initial step or phase underpins the clarification of the problem and strategic focus. For CoPAI, this started with the observation of an increase in academic misconduct at the NWU (and in HE in general) since the inception of the pandemic. This was evident through an analysis of student assessment records where the NWU embarked on an institutional thematic review that encapsulated both the perceptions of staff and students on assessment practices since the inception of the pandemic in March 2020. The impact of the pandemic was visible through increased pass rates, decreased drop-out rates, and a heightened concern about the increased academic misconduct. This increased academic misconduct posed various challenges to the NWU, especially the integrity of the institution and its qualifications. Following the affirmative nature of appreciative inquiry, the identified problem, i.e., increased academic misconduct, and strategic focus to increase academic integrity was framed in a positive context, approach, and affirmative topic of choice, namely “The enhancement of Academic Integrity through a Community of Practice at the NWU”.

**Fig. 2:**
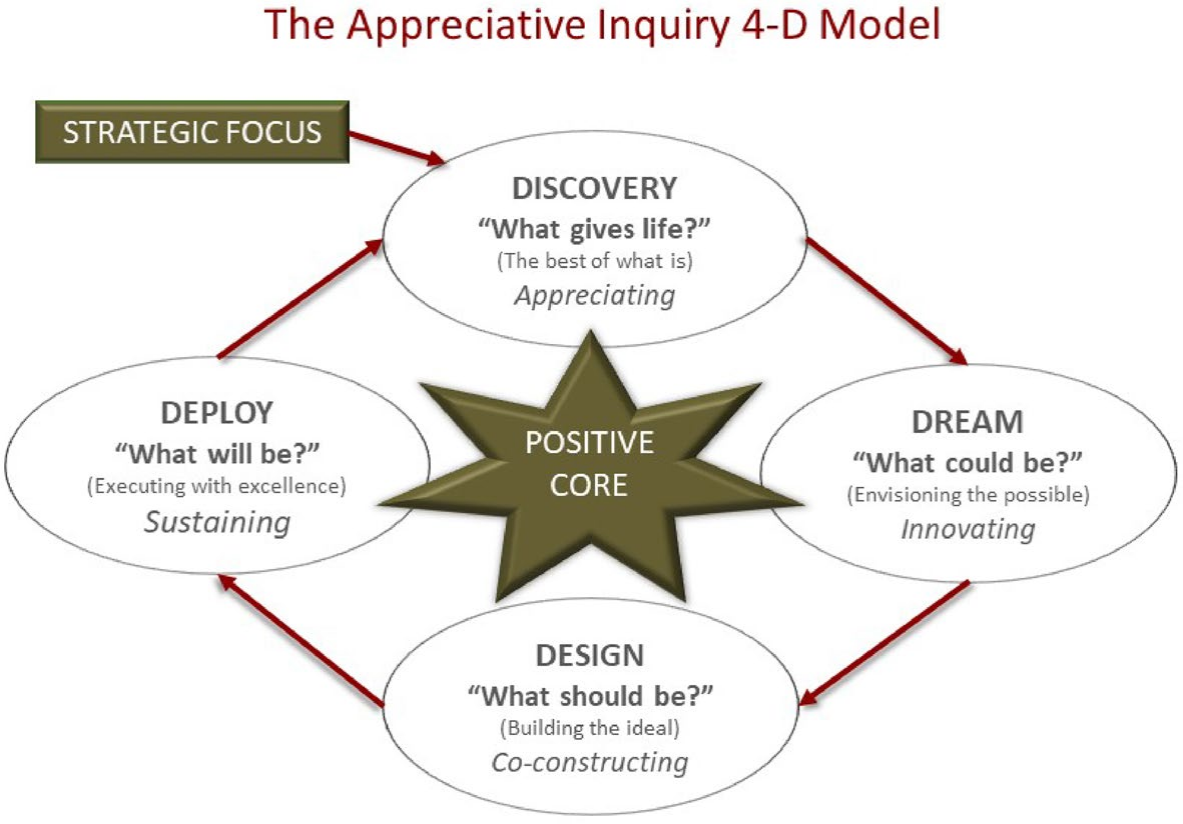
(Source: Benedictine University, 2017)

This first step in an appreciative inquiry methodological approach, namely ‘clarification’, determines the implementation and functioning of appreciative inquiry as a methodology, as embedded in the 4D model. For the CoPAI, clarification of the purpose, strategy and a holistic approach determined the implementations and functioning of the CoPAI. The 4D model explains the relevant phases of an appreciative inquiry process to drive positive transformation in the ambit of the contextualised focus of inquiry. The 4D model is depicted in Fig. 2 and explained for the CoPAI context below.

The four different phases of this appreciative inquiry 4D model can be described as follows:

- *1. Discovery*. The first phase involves discovery, which underpins the appreciation concept. During this phase, the CoPAI explored “the best of what is” by sharing best practices through hosting discussion forums. It is an active inquiry phase to uncover strengths, creating a positive mindset and vocabulary, and moving away from deficit-focused thinking. Specific topics were chosen from themes emerging from this active inquiry process for further inquiry. This approach was followed by CoPAI in arranging different topics for the various discussion forums. As a community of practice, our identity was defined by a shared domain of interest (enhancing AI) where participants formed relationships, engaged in joint activities and discussions (e.g., forums), helped each other, shared information, and best practices, and learned from one another and/or together.
  This took place on a practical level during the first CoPAI forum where some shortcomings in the academic integrity discourse from a teaching and learning perspective were discovered, and then explored by participants to find the best of what is by sharing best practices.
  - This included the importance of disciplinary literacies and the specific questions around transition from high school into university that emerge out of that, and its relation to pedagogical content knowledge. We explored together what kinds of knowers (knowledgeable people) we want to produce in a specific discipline, how we generate knowledge that will be transferred to students in ways that observe the disciplinary regulations or conventions within the discipline itself, and what constitutes knowing in that particular discipline.
  - It was also explored how first-generation students are possibly placed at risk if we fail to developmental opportunities to both staff and students to elevate the burden of academic misconduct. In this regard, the idea of creating effective learning communities, places and spaces for students was discussed, as well as student societies to create an atmosphere that will create students who “have no need to cheat” or “have no need to plagiarise”.
  - It was also explored how a data-driven approach concerning whom we teach and assess, and what prior knowledge and experiences students bring into classrooms based on their socio-cultural diversity, can be adopted.
  - Two other aspects were also explored, namely the importance of incorporating the student voice in curriculum design, and subsequently also in this CoPAI, and the important role the Student Judicial Services (SJS) office can play at a university.
    During this first CoPAI forum, academics expressed their need to be part of CoPAI in the context of the complexity of dealing with academic misconduct, to improve their own practices of academic integrity. They also expressed the need for more synergy between support departments and faculties. Participants furthermore acknowledged that the CoPAI could be the ideal space for enabling more intra-institutional collaboration. Colleagues from the Centre for Teaching and Learning (CTL) also alluded to the importance of a quality-driven and data-driven approach to academic integrity. Issues of students’ linguistic capital were raised in ensuring that students have a disciplinary voice through epistemological access to the discipline. CTL further elaborated on the importance of supporting both staff and students, curriculum development, and the design of assessments in driving academic integrity.
- *2. Dream*. The dream phase envisions the potential positive future, what is being called for or ‘what might be?’ In the CoPAI context, the dream is to enhance AI through CoPAI. This phase emphasises innovation for enhancing AI as derived from multiple perspectives, opinions, and understanding as evident from the attendance and engagement of the CoPAI members. In the context of the strategic focus of inquiry, i.e., to enhance AI, this phase unlocks creativity and constructive visions and possibilities to realise the dream. Members of CoPAI co-created positive outcomes for a preferred future at our institution following a holistic approach. Such positive outcomes can be observed at each CoPAI forum that was held, but forum two can by highlighted in this regard.
  With the second CoPAI forum we started to dream how academic integrity, and the academic reputation of the university can be protected by every staff member and student. We realised we all must take ownership in upholding, maintaining, and managing academic integrity. This dream became part of our holistic dream and approach that combines 1) an educative focus on academic integrity policy dissemination, 2) a robust and fair process for dealing with breaches, and 3) using data from reporting to improve teaching and learning. In this regard was emphasised that there should be consistent and effective institutional policies and practices that focus on educative measures, different types of policy breach applicable penalties, and clear processes for investigating breaches. All of these became a task or the focus of the CoPAI combined with institutional responsibility. The need was also identified that CoPAI should engage with and empower lecturers on the one hand, and students on the other. Students should, for example, be as knowledgeable as possible of the nature of academic integrity decisions and the possible consequences. Some other possibilities were shared at this forum, namely:
  - An ipsative approach to assessment which emphasised that academic integrity might be improved through apt assessment designs and the provision of constructive feedback and understanding the current student profile and some prevalent and scientifically proven challenges we are facing of the type of student that we are teaching nowadays.
  - An understanding of millennial students’ views about academic honesty and dishonesty that may provide a lens through which to view and address academic dishonesty and may then also be used to devise strategies to support the ethical development of students. Various remedial strategies were proposed, and the discussion was concluded by the contribution that the educational purpose of the university is not to prepare students for their role in the future workplace, but rather to bring students into a transformational relationship with knowledge that challenges their sense of who they are and what they can do in the world.
- *3. Design and delivery*. This phase entails the continuous design, delivery, and co-creation of high-impact strategies that could move the organisation forward in a creative and decisive manner. In the CoPAI context, this phase encapsulates the various initiatives in which CoPAI strives to engage towards enhancing academic integrity at the institution. This phase is evident from the third CoPAI discussion forum. With the third CoPAI forum, the emphasis was for example on sharing practical solutions for issues related to academic integrity, with special reference to assessments in the online environment with large classes. Several presenters focused on the importance of rethinking how we assess, and how changing the assessments can directly influence academic integrity. The design of authentic assessments was proposed as an alternative, whilst allowing room for creativity and making assessments more engaging.
  As part of the design, delivery, and co-creation of high-impact strategies this from also focused on the different perceptions and definitions lecturers have of academic dishonesty, which are often revealed in how a Turnitin report is interpreted (i.e., (mis)perceptions regarding Turnitin similarity indices). Closely related to the former is the notion of perceptions regarding intentionality (i.e., when a student is culpable or acted with intent). Therefore, when considering academic integrity and possible dishonesty or misconduct, it is important and even vital to distinguish between dishonesty, PAWP (poor academic writing practices), and criminality, and to separate the concepts.
  Given the above, it is not surprising that members of CoPAI expressed a need for the training of lecturers regarding assessment design, to create more appropriate assessments. At the same time, lecturers also reported that students were scared of possibly plagiarising and therefore needed to be better informed on this issue.
  The fourth CoPAI forum continued the design and delivery aspects of the 4D approach. It specifically focusses on matters relating to the NWU Policy on Academic Integrity and procedures outlined therein to ensure that all academic activities are conducted with integrity. This discussion was complemented with inputs from the NWU Student Judicial Services (SJS) and feedback from the Student CoPAI forum hosted earlier.
  The holistic approach of the NWU Policy on Academic Integrity policy and the clear guidelines outlined in it to ensure that academic integrity is infused throughout all academic processes were consequently discussed. The primary concern among the participants included the following:
  - How should we go about creating awareness of the policy among students (specifically when, what, where, and by whom)?
  - How can SJS’s capacity to process a vast array of cases be improved and how can the reporting process be streamlined?
  - How should staff identify and deal with cases regarding academic misconduct amongst students who enrol for courses cross-faculty?
  - How can record keeping be improved, especially where record-keeping of cases is dealt with internally by faculties and how can this information be made accessible inter-faculty?
  - What is the difference between academic dishonesty and poor academic writing practice (PAWP)^7^?
    The fifth CoPAI forum also focused on strategies that could move the university forward in a creative and decisive manner. The focus was on the compulsory first year courses in Academic Literacy because within these modules, a specific method of dealing with plagiarism has been developed that considers the need for a responsible, consistent, but – above all – pedagogical approach. This method was also explained to staff who were not so well informed about this.
    Actions resulting from the fifth forum discussion included a request from the Student Judiciary Office staff member for access to all the study materials (online and in print) used in Academic Literacy. Staff at Academic Literacy also committed to provide information to all other academic staff members on the methods, content and time frames used by the subject group Academic Literacy. This should enable other staff members to reinforce the material and to refer students back to the information in Academic Literacy. In addition, it would give lecturers insight into what they can reasonably expect learners to know and be able to do.
- *4. Deploy or destiny*. This final phase constructs futures through innovation and action. It is particularly during this phase where organisational cultures are changed through insights gained by following the appreciative inquiry process.

A good example of how an effort was made through CoPAI to change organisational cultures is found in the discussion of the first student CoPAI forum. Student involvement was part of CoPAI’s strategy from the beginning, with its focus on lecturers, the institution, and students. After the first three CoPAI forums, to which students were also invited, it became clear that a dedicated student constituent was needed for CoPAI in order to give students more opportunity and freedom to share their ideas and raise their concerns.

At this forum, the director of the Centre for Teaching and Learning was the introductory speaker, and he gave an overview of the importance and place of assessments in formal education. One reason for student dishonesty in the South African context, according to him, is the fact that many of them do not acquire reading and writing skills at a young age. Lecturers assume that all students have obtained a level of literacy development, as in Western middle-class societies, whereas the majority of students did not grow up with bedtime stories, books, the skills to anticipate in a written text what will happen next (the logical structure and expectation of texts), or the ability to read independently. Lecturers made the wrong (Western, middle-class) assumption that students can learn from reading, and students are then evaluated on this basis. However, students are not explicitly taught to read, learn, and understand, and this leads to underperformance and eventually to academic misconduct like plagiarism.

The SJS representatives explained the legal procedures that are followed when students are reported for academic misconduct. The students engaged very enthusiastically on this point, and it was clear that there was misunderstanding and a lack of information on the students’ side, which led to their distrust of SJS. Perhaps the most valuable outcome of this forum was that students and SJS could have an open, non-threatening conversation about their perceptions and to clarify many of the misinformation that existed. The students had many questions about what academic misconduct entails, and group work was indicated as problematic in this regard. The need to clarify and educate students on the different forms of academic misconduct became very clear.

The consequent steps of action to follow from this forum was the need to develop SOPs for faculties, more exchange of information between SJS and students, training of all stakeholders about procedures and about what academic misconduct entails, and a prioritisation of helping students to read, learn and understand better from written texts. All of these aspects are still priorities of CoPAI and evident on the action list, but the advantage of discussing such aspects at these forums is embraced in a collaborate approach in deciding together on the necessary steps to be taken.

## Conclusion

The concrete outcomes of the different activities of CoPAI at NWU are discussed as part of the conclusion of the article. The main activities of the NWU CoPAI since its inception refers to the organising and hosting of the various discussion forums. The forums were the basis from where other activities were planned and implemented. The forums served in this sense the purpose of Dream and Design phase of the appreciative inquiry approach, but from these forums Design and Deploy phase activities also followed. Without repeating what has been summarised above in the analysis of the CoPAI forums, we highlight some of the main concrete outcomes of CoPAI at the NWU below.

With reference to the purpose of CoPAI, we succeeded in:

- Creating opportunities (through the different forums) where lecturers, students and support departments could share best practices in a free and open way. This also took place on the website and Facebook sites of CoPAI, as well as through email exchanges between participants of CoPAI (colleagues from different faculties who have met through CoPAI).
- Delivering some practical outputs and guidelines, as well as research outputs on AI. The practical outputs and guidelines were shared in conversation on assessment and curriculum design, large classes online assessments, discussion on plagiarism and Turnitin reports, etc. The most practical and urgent output is, however, the development of Standard Operating Procedures (SOP) for the teaching and learning environment that is currently (2022) being developed by a CoPAI team (discussed below).
- Collaborating with national and international scholars through sharing of best practices, and by inviting them as speakers to the CoPAI forums. This happened in a very natural way because the Zoom link for the forums were forwarded to colleagues from other universities in South Africa who regularly attended our forums and participated in it. At our first CoPAI forum of 2022, which is not analysed in the discussion above, the invited speakers were both from the European Network for Academic Integrity (ENAI) – one from the UK and the other from Sweden. The writing of this article is also aimed at sharing best practices to national and international scholars.
- Establishing a CoPAI identity through various marketing strategies. As mentioned before, CoPAI has established its own CoPAI website and a CoPAI Facebook page which enhanced collaboration and engagement amongst its community members. An initial report in the university newsletter was also published on the NWU webpage. With every CoPAI forum its visibility grows, and it has become a well-established CoP at the NWU by now. Appreciation for its work is for example often mentioned at Teaching and Learning Committees and on the Senate of the NWU.

Many CoPAI forums led to an exchange of information afterwards between participants and between different support departments and faculties. For example, the learning material of the Academic Literacy modules were sent to the SJS to incorporate it into their procedures. Another direct outcome of one of the CoPAI forums is the development of a short learning programme (SLP) titled “Introduction to Academic Integrity”, as well as online professional development resources. Aspects that are considered for inclusion in the SLP refers to (i) a shared definition of academic integrity at the NWU, (ii) reasons why students plagiarise and cheat, (iii) cognition and its implications for academic integrity, (iv) assessment design and practice, (v) moving beyond academic misconduct, and (vi) the vital intersection between academic integrity and research integrity.

The forums also helped to produce a sense of empowerment of the lecturers – something that is of course difficult to measure, but continuously reported by the participants. The student forum helped for example to eliminate distrust between students and SJS and created an environment where more constructive discussions can follow in future.

As mentioned above, one of the main outcomes of the CoPAI forums and activities is the development of standard operating procedures (SOPs) from a TLA perspective for academic integrity at the NWU. The need for such SOPs was consistently communicated by all CoPAI members at various forums. An inter-faculty task team, under the leadership of CoPAI, has since been established and are already working on these SOPs.

It is clear from these discussions and outcomes that the CoPAI at the NWU helped and contributed in several ways to enhance the academic integrity at this institution. This testifies of our institution to remain relevant, responsive, and agile within an overarching transformational framework towards student success when faced with disruptions in the higher education (HE) landscape It is also clear that this is an ongoing task and that pertinent shortcomings regarding AI processes (e.g., development of SOPs) at this institution should be prioritised and addressed. The CoPAI at NWU succeeded here in identifying some of the most crucial needs for enhancing AI further.

The main contribution of this article to the field of academic integrity, is its substantial indication of the importance, value, and relevance of a CoPAI for the enhancement of AI at HE institutions. The usefulness of this CoPAI is emphasized through its holistic approach including the valorisation of institutional aspects, the engagement and empowerment of lecturers, and the engagement and empowerment of students. The authors recommend that CoPAI at NWU in South Africa might also be applicable at other institutions of HE globally to follow.

Further research suggested for a CoPAI includes how to efficaciously maintain a successful CoP towards collaborative knowledge creation within the ambit of time constraints and workload demands as it remains voluntary and organic in nature. The question is also how to implement it at different HE institutions which have all their own unique culture and context, but at least some aspect of the establishment and value of the NWU CoPAI should be translatable in the South African and global context.

## Data Availability

Not applicable.

## Abbreviations

AI: Academic Integrity
CoP: Community of Practice
CoPAI: Community of Practice for Academic Integrity
CTL: Centre for Teaching and Learning
ENAI: European Network for Academic Integrity
FTLS: Faculty Teaching and Learning Support
HE: Higher Education
HEI: Higher Education Institutes
IOAs: Interactive Oral Assessments
LMS: Learning Management System
NWU: North-West University
PAWP: Poor Academic Writing Practices
SJS: Student Judicial Services
SLP: Short Learning Programme
SOP: Standard Operation Procedures
SoTL: Scholarship of Teaching and Learning
TLA: Teaching, learning and assessment

## Footnotes

1 This conference was organised by the European Network for Academic Integrity and a wide variety of themes were discussed. For more detail and for the Book of Abstracts, see https://academicintegrity.eu/conference/enai-conference-2021/

2 This special published the proceedings from the second Canadian Symposium on Academic Integrity (CSAI) 2021 virtually hosted by Thompson Rivers University, Kamloops, British Columbia, Canada. See https://journalhosting.ucalgary.ca/index.php/ai/issue/view/5302

3
http://services.nwu.ac.za/copai

4
https://www.facebook.com/NWU-Community-of-Practice-Academic-Integrity-101369588885010

5
https://www.nwu.ac.za/sites/www.nwu.ac.za/files/files/i-media/eng-eish-202106/integrity.html

6
https://www.nwu.ac.za/sites/www.nwu.ac.za/files/files/i-governance-management/policy/2021%20Update/2P_2.4.3.2/2P_2.4.3.2_2021_e.pdf

7 The Annexure 3 of the Policy (2021) defines PAWP as the involvement in “possible collaboration or poor citation practice in which evidence is obvious that (i) the researcher/student did not appreciate the rules for academic writing, or (ii) where the extent of copied material is considered to be of minor impact or slight copying”. When a case is classified as PAWP, remedial action would be followed without proceeding to disciplinary action, but follow-up transgressions will be taken if remedial action was not fully appreciated by the transgressor.
